# Cannabidiol potentiates phenobarbital effects in the control of pentylenetetrazole (PTZ)-induced epileptic seizures in neonate rats

**DOI:** 10.3389/fped.2025.1673345

**Published:** 2025-11-10

**Authors:** Lillian Soares Pinto, Matheus Silva de Oliveira, Giovanna Bruno Borges, Olagide Wagner de Castro, Fabrício de Araújo Moreira, Victor Rodrigues Santos

**Affiliations:** 1 Department of Morphology, Institute of Biological Sciences, Federal University of Minas Gerais, Belo Horizonte, Brazil; 2 Cell Biology Graduate Program, Institute of Biological Sciences, Federal University of Minas Gerais, Belo Horizonte, Brazil; 3 Department of Physiology, Institute of Biological Sciences, Federal University of Alagoas, Maceió, Brazil; 4 Department of Pharmacology, Institute of Biological Sciences, Federal University of Minas Gerais, Belo Horizonte, Brazil

**Keywords:** epilepsy, endocannabinoid system, cannabidiol, phenobarbital, neonatal period

## Abstract

Epilepsy is characterized by the predisposition to epileptic seizures resulting from neuronal hyperexcitability and hypersynchrony. Seizure management consists primarily of the long-term use of antiseizure drugs, such as phenobarbital (PB). However, many patients, especially neonates, exhibit resistance to PB and can suffer adverse effects, including abnormal neuronal apoptosis. Cannabidiol (CBD), a non-psychotomimetic phytocannabinoid CBD has demonstrated efficacy in attenuating epileptic seizures. However, its interaction with PB remains largely unexplored. This study investigated the potentiation effect of CBD on PB in a neonatal pentylenetetrazole (PTZ)-induced seizure model. Ten-day-old (P10) Wistar rats were intraperitoneally pretreated with PB (3, 10, 30, 50, or 75 mg/kg) and/or CBD (3, 30, 100, or 200 mg/kg). After 60 min, seizures were induced by subcutaneous administration of PTZ (100 mg/kg), and seizure latency, duration, and severity were subsequently assessed. Low doses of CBD (3 and 30 mg/kg) exhibited limited efficacy when administered alone, while higher doses (100 and 200 mg/kg) modestly attenuated PTZ-induced seizures. However, CBD (30, 100, or 200 mg/kg) significantly enhanced the efficacy of a subeffective dose of PB (10 mg/kg). These results indicate a dose-dependent potentiation by CBD of PB effects, supporting the potential of CBD as an adjunct therapy for neonatal seizures.

## 1 Introduction

Epilepsy is a chronic neurological disorder characterized by a predisposition to recurrent seizures and their associated long-term consequences (1). Globally, it affects over 60 million individuals, posing a significant public health burden, with more than 11 million cases occurring in children under 15 years of age (2). Seizures result from excessive or abnormal synchronous neuronal activity. They are particularly prevalent in neonates, where they are often linked to insults to the central nervous system (CNS), cortical malformations, inborn errors of metabolism, or genetic epileptic syndromes (3). The International League Against Epilepsy (ILAE) classifies seizures by onset (focal, generalized, or unknown) and further categorizes them by motor involvement and awareness (4).

Antiseizure medications (ASMs) modulate neuronal excitability by targeting ion channels and neurotransmitter systems, primarily glutamatergic and GABAergic pathways (5). However, a substantial challenge in epilepsy treatment is pharmacoresistance, observed in approximately one-third of patients (6–8). Neonates are particularly vulnerable to pharmacoresistance due to their immature brain development (9, 10). Phenobarbital (PB), a first-generation ASM, remains the standard treatment for neonatal seizures (3). As the main mechanism of action, the PB primarily increases the activation of the GABA-A receptor, promoting the influx of chloride ions and greater inhibition in neural circuitry (11). Despite its widespread use, PB is associated with neurotoxicity, cognitive impairments, and widespread transcriptomic changes. The neurotoxicity of PB is evidenced mainly in animal models, where therapeutic doses, such as 75 mg/kg, usually cause neuronal apoptosis in the developing brain of rodents (11–15). Clinical data underscore these concerns, reporting cognitive deficits in pediatric patients treated with PB, thereby highlighting the urgent need for safer therapeutic alternatives (16–19). Furthermore, drugs such as valproate, PB, phenytoin, diazepam, clonazepam, lamotrigine, vigabatrin, as well as ethanol and anesthetic agents, are known to induce acute neurotoxicity, including neuronal apoptosis, after a single exposure during a critical postnatal brain development window in rodents, peaking around postnatal day 7 (P7) (12, 20). This P7 timepoint in rodents models a period spanning from the third trimester through early infancy in humans, corresponding to the peak of the brain growth spurt (21).

Cannabidiol (CBD), a non-psychotomimetic cannabinoid derived from *Cannabis sativa*, modulates neuronal excitability through various targets, including CB1/CB2 receptors, TRPV1, GPR55, and PPARγ (22–29). Its mechanisms involve inhibiting glutamate release, modulating adenosine reuptake, and blocking sodium channels (24, 26, 30). Beyond its direct antiseizure properties, CBD also exhibits neuroprotective and anti-inflammatory effects by reducing oxidative stress and inflammatory cytokines while promoting anti-inflammatory mediators. CBD is currently FDA-approved for refractory epilepsy syndromes such as Dravet and Lennox-Gastaut syndromes. Preclinical data further suggest that CBD's mechanisms of action may also be effective in controlling neonatal seizures (31–36).

Given the known properties of PB and the emerging evidence for CBD, combining CBD with PB may offer a strategy to enhance seizure control while potentially minimizing the adverse effects associated with PB monotherapy. This study investigates the potentiation exercised by CBD of PB effects in a neonatal rat model of Pentylenetetrazole (PTZ)-induced seizures. By evaluating the combined properties of these two compounds, we aim to provide insights into developing safer and more effective treatment strategies for neonatal epilepsy (37–41).

## 2 Methods

### 2.1 Animals

Adult male and female Wistar rats were obtained from the local animal facility (CEBIO) and housed in the Department of Morphology at the Institute of Biological Sciences (ICB), Federal University of Minas Gerais (UFMG). Animals were maintained under controlled conditions, including a temperature of approximately 21 °C and a 12 h light/dark cycle (07:00–19:00), with food and water available *ad libitum*.

Following breeding, male and female Wistar pups were obtained and used for experimental procedures approved by UFMG's Ethics Committee for Animal Research (CEUA—226/2022). A total of 157 neonate rats were used for this study, *n* = 157, as approved by CEUA. Pups were treated on postnatal day 10 (P10), with P0 defined as the day of birth. During the experiments, P10 Wistar pups were pre-treated with drugs and subsequently exposed to a Pentylenetetrazole (PTZ)-induced seizure model 60 min after drug administration. Treatments were balanced within groups and across litters, ensuring an approximately equal distribution of males and females. All experimental procedures were conducted during the light phase.

### 2.2 Drug administration

Phenobarbital (PB, “Fenocris®”) was dissolved in dimethyl sulfoxide (DMSO) and administered intraperitoneally (i.p.) at a volume of 10 mL/kg, at the doses of 3, 10, 30, 50 and 75 mg/kg (13, 42). Cannabidiol (CBD, THC Pharma, >99% purity as certified by the manufacturer) was diluted in a vehicle solution containing 2% Tween 20 and 0.9% saline and was administered i.p. at a volume of 10 mL/kg, delivering doses of 3, 30, 100, and 200 mg/kg (43, 44). The CBD that was used in this study is natural and was extracted from the plant whose species is *Cannabis sativa*. All drug solutions were prepared immediately before administration to ensure optimal pharmacokinetic properties. The higher CBD doses (100 and 200 mg/kg) were chosen based on previous studies demonstrating that antiseizure effects in neonatal rodents often occur only at high systemic concentrations (41, 45). These doses are near but below the known toxicity threshold for CBD in rodents (15) and allow evaluation of dose-response relationships. While such concentrations may not directly translate to human clinical use, they provide mechanistic insights into CBD's potential as an adjunct in settings of pharmacoresistance.

### 2.3 Pentylenetetrazole-induced seizure model

Pentylenetetrazole (PTZ, “Sigma®”) was dissolved in 0.9% saline and administered subcutaneously (s.c.) at a dose of 100 mg/kg. Neonatal Wistar rats were removed from their cages, weighed, numbered, and sexed before drug administration. Animals were pre-treated with CBD and PB, separately or combined at various doses, although the PB at the dose of 3 mg/kg was administered exclusively in combination with CBD. The antiseizure drugs were administered 60 min before PTZ. This time point was selected based on previously established pharmacokinetic data on CBD and the PB action in neonatal animals (41, 43, 46). When CBD and PB were administered in combination, there was an interval of minutes between their administration (45, 47–51). After PTZ injection, pups were placed in transparent acrylic cages without lids and filmed for 15 min to assess seizure latency, duration, and intensity.

The PTZ model was employed to induce seizures in P10 rats due to its well-established and reproducible characteristics, as well as its widely recognized mechanism of action (52, 53). When administered during the neonatal period, PTZ is associated with low systemic toxicity and reduced mortality rates (46, 54, 55). This model enables the assessment of seizure parameters—including latency, duration, and intensity—within a relatively short timeframe. Moreover, similar to the maximal electroshock seizure (MES) model, PTZ is considered a reliable approach for evaluating the efficacy of candidate anticonvulsant compounds (41, 54).

### 2.4 Behavioral assessment of seizures

P10 rats were chosen as this age corresponds to a period spanning the late third trimester through early infancy in humans (56). This developmental phase is characterized by increased synaptogenesis (21, 57), analogous to 9–12 months of human brain development (56). CBD was administered 60 min prior to PTZ injection, following a time course previously demonstrated to be effective in mitigating PTZ-induced seizures in adult rats at a comparable dose range (41, 43, 44, 51, 54, 58–61).

To maintain body temperature, animals were returned to their dam until immediately before PT*Z* testing. Following PTZ injection, they were placed in transparent plexiglass observation chambers, where seizure activity was monitored. Latency to seizure onset and seizure incidence were recorded by treatment-blind observers (L.S.P.; M.S.O. and/or G.B.B). Observations continued for 30 min post-PTZ administration.

#### 2.4.1 Seizure scoring

Seizure severity and latency to onset were documented. Seizure duration sum was recorded, as seizures induced by this PTZ dose in rat pups of this age typically persisted throughout the observation period. Seizure severity was assessed using the rating scale established by Kubová; Mares (48), ensuring consistency with previous studies conducted by our lab (49) and others (42, 48, 62, 63). The scoring criteria were as follows:

- **0:** No observable behavioral changes
- **1:** Myoclonic jerks
- **2:** Unilateral clonus, chewing/shuffling, Straub tail
- **3:** Facial and forelimb clonus
- **4:** Running/bouncing clonus with loss of righting reflex
- **5:** Running/bouncing clonus with loss of righting reflex and tonic extension (equivalent to the “complete major seizure” described by (48).

Mean latencies were reported only for groups in which at least 50% of animals exhibited seizures.

### 2.5 Perfusion

On postnatal day (P) 10, pups were treated with drugs and euthanized 2 h later through a combination of Ketamine with Xylazine (Abbott) and 0.9% saline solution, whose ratio was 2:1:1 injected intraperitoneally (i.p.) at a dose of 100 mL/kg. After that, the animals were transcardially perfused with the aid of an injection applied to the left ventricle containing 15 mL of 1% phosphate-buffered saline solution (PBS) with the purpose of cleaning the blood. Then, an injection was applied to the same site containing 20 mL of paraformaldehyde (4% PFA in PBS, pH = 7.4) to fix the brain tissue (64).

After perfusion, the brains were extracted and placed in fixative (PFA 4%) overnight, and transferred to sucrose solutions with PBS in the percentages 10%, then 20% and then 30%, with a 24 h stay in each solution to ensure cryoprotection. After at least 48 h, the brains were frozen in 99% isopentane (Vetec) and dry ice (−65  °C), then stored at −80 °C until they were cut in cryostat (Leica CM1860 UV). Perfusion and storage techniques were necessary so that it was later possible to perform histochemical and immunohistochemical techniques with the brains collected from P10 rats.

### 2.6 Statistical analysis

Statistical analyses were performed using GraphPad Prism 8 (GraphPad Software, La Jolla, CA). Seizure latency and duration data were assessed for normality (D'Agostino and Pearson test) and, where nonparametric, were analyzed using the Kruskal–Wallis test followed by Dunn's multiple comparisons *post hoc* test. Proportions of seizure severity were compared using contingency table analysis. All tests were one-tailed, considering the directional hypothesis of CBD efficacy, and *p* < 0.05 was considered statistically significant.

## 3 Results

### 3.1 Antiseizure efficacy of phenobarbital (PB) in PTZ-induced seizures in neonatal rats

**Figure 1:**
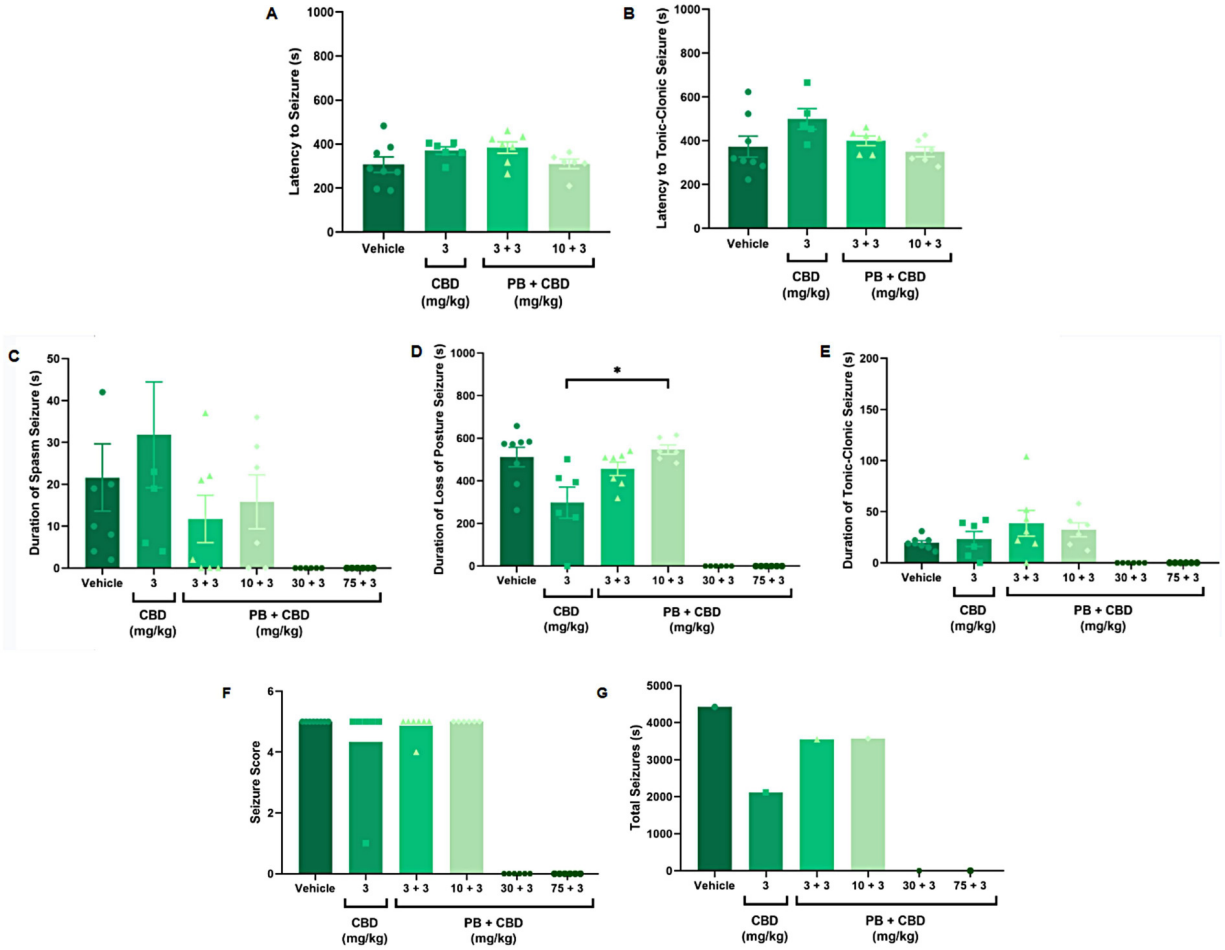
Antiseizure effects of low-dose cannabidiol (CBD, 3 mg/kg), alone or in combination with phenobarbital (PB; 3, 10, 30, or 75 mg/kg), in a PTZ-induced seizure model in P10 rats. **(A,B)** Mean latency to seizure onset and to the most severe seizure event; *n* = 8, 6, 7, 6 **(A,B)**. **(C–E)** Mean duration of spasms, loss of righting reflex (postural seizures), and tonic-clonic seizures; *n* = 8, 6, 7, 6, 6, 6 **(C–E)**. **(F)** Mean seizure severity score; *n* = 8, 6, 7, 6, 6, 6. **(G)** Total seizure duration: *n* = 8, 6, 7, 6, 6, 6. CBD at 3 mg/kg alone modestly reduced the duration of postural seizures **(D)** and total seizure time **(G)** but did not significantly affect seizure severity **(F)** Combinations of low-dose CBD with subeffective PB (3 or 10 mg/kg) did not substantially improve seizure parameters. Data were analyzed using the Kruskal–Wallis test; *p* < 0.05 was considered statistically significant (*p = 0.0306 in panel **D**).

**Figure 2:**
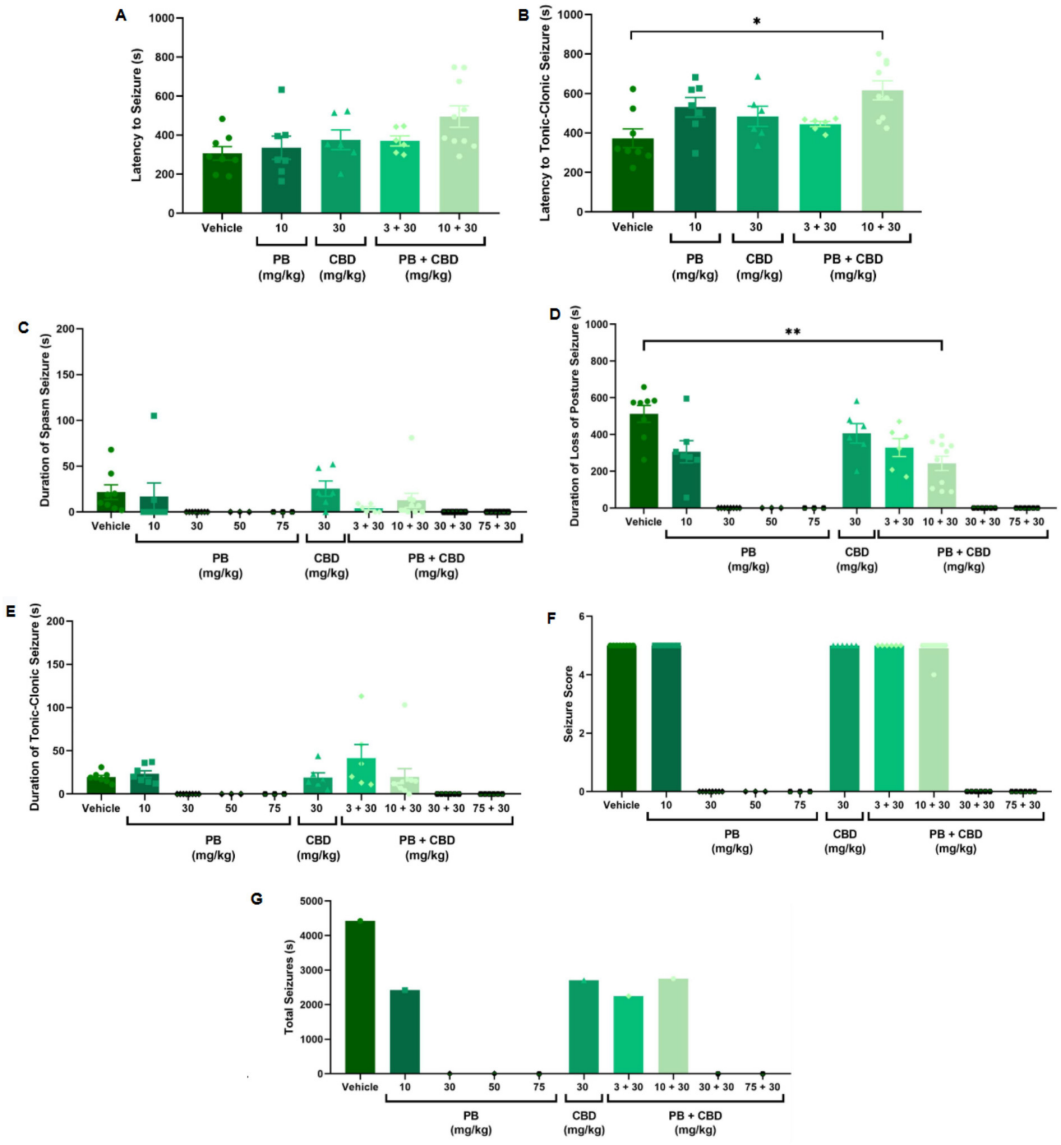
Antiseizure effects of cannabidiol (CBD; 30 mg/kg), alone or in combination with phenobarbital (PB; 3, 10, 30, or 75 mg/kg), in the PTZ-induced seizure model in P10 rats. **(A,B)** Latency to seizure onset and to the most severe (tonic-clonic) seizure; *n* = 8, 7, 6, 6, 10 **(A,B)**. **(C–E)** Mean duration of spasms, loss of righting reflex (postural seizures), and tonic-clonic seizures; *n* = 8, 7, 8, 3, 3, 6, 6, 10, 6, 6 **(C–E)**. **(F)** Seizure severity score; *n* = 8, 7, 8, 3, 3, 6, 6, 10, 6, 6. **(G)** Total seizure duration; *n* = 8, 7, 8, 3, 3, 6, 6, 10, 6, 6. Co-administration of 10 mg/kg PB with 30 mg/kg CBD significantly increased the latency to tonic-clonic seizure onset **(B)** and reduced the duration of postural seizures **(D)**. However, seizure severity **(F)** and total seizure time **(G)** remained similar across groups. Data are presented as mean ± SEM and were analyzed using the Kruskal–Wallis test. *p* < 0.05 was considered statistically significant (*p = 0.0104, **p = 0.0097 in respective panels).

**Figure 3:**
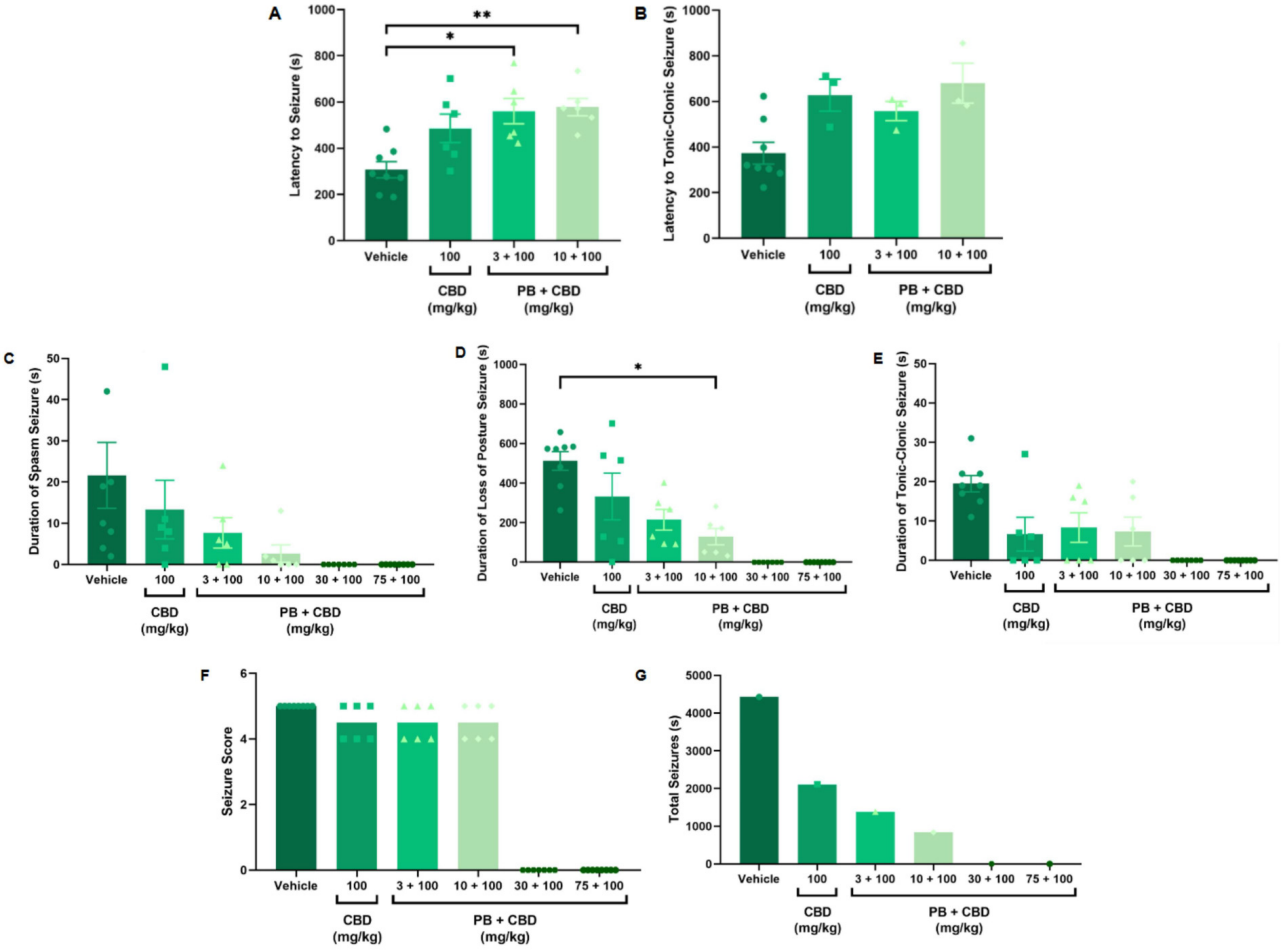
Antiseizure effects of cannabidiol (CBD, 100 mg/kg) alone or in combination with phenobarbital (PB; 3, 10, 30, or 75 mg/kg) in the PTZ-induced seizure model in P10 rats. **(A,B)** Latency to seizure onset and to the most severe (tonic-clonic) seizure; *n* = 8, 6, 6, 6 **(A,B)**. **(C–E)** Duration of spasms, loss of righting reflex (postural seizures), and tonic-clonic seizures; *n* = 8, 6, 6, 6, 7, 8 **(C–E)**. **(F)** Seizure severity score; *n* = 8, 6, 6, 6, 7, 8. **(G)** Total seizure duration; *n* = 8, 6, 6, 6, 7, 8. Co-administration of 100 mg/kg CBD with either 3 or 10 mg/kg PB significantly increased latency to seizure onset **(A,B)**, and the 10 mg/kg PB + 100 mg/kg CBD combination significantly reduced the duration of postural seizures **(D)**. However, seizure severity **(F)** was not significantly altered across groups. Notably, the group receiving CBD alone (100 mg/kg) exhibited a longer total seizure duration compared to CBD + PB combinations **(G)**, excluding the control group. Statistical analysis was performed using the Kruskal–Wallis test. *p* < 0.05 was considered statistically significant (*p = 0.0206, **p = 0.0092, *p = 0.0117 in relevant panels).

**Figure 4:**
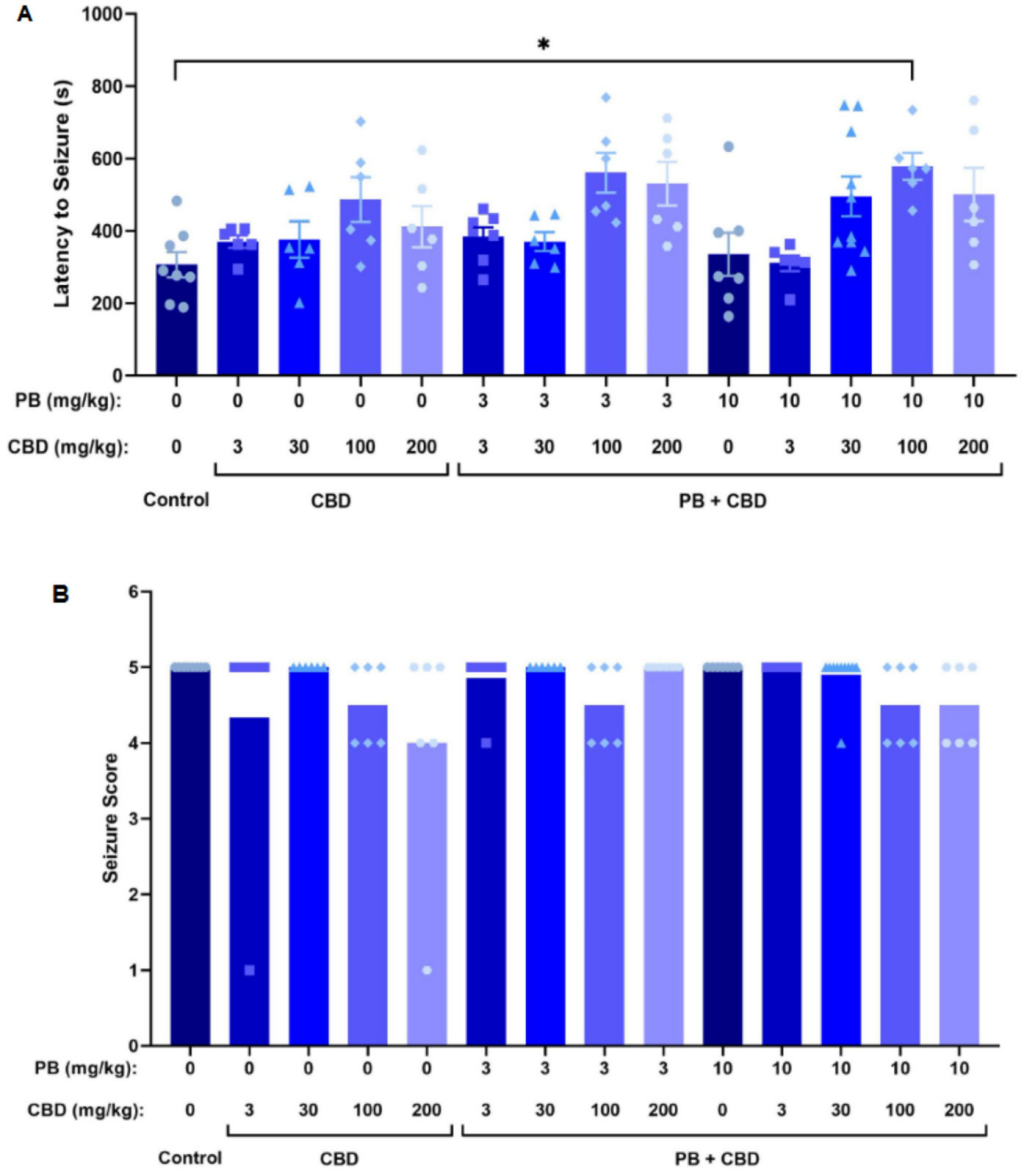
Antiseizure effects of cannabidiol (CBD) and phenobarbital (PB), administered alone or in combination, during PTZ-induced seizures in P10 rats. **(A)** Mean latency to seizure onset as a function of PB and CBD doses; *n* = 8, 6, 6, 6, 6, 7, 6, 6, 6, 7, 6, 10, 6, 6. **(B)** Mean seizure severity scores across treatment groups; *n* = 8, 6, 6, 6, 6, 7, 6, 6, 6, 7, 6, 10, 6, 6. Co-administration of CBD with PB significantly increased seizure latency in certain groups **(A)**, although no significant differences in seizure severity were observed **(B)**. Data were analyzed using the Kruskal–Wallis test. *p* < 0.05 was considered statistically significant (*p = 0.0268).

**Figure 5:**
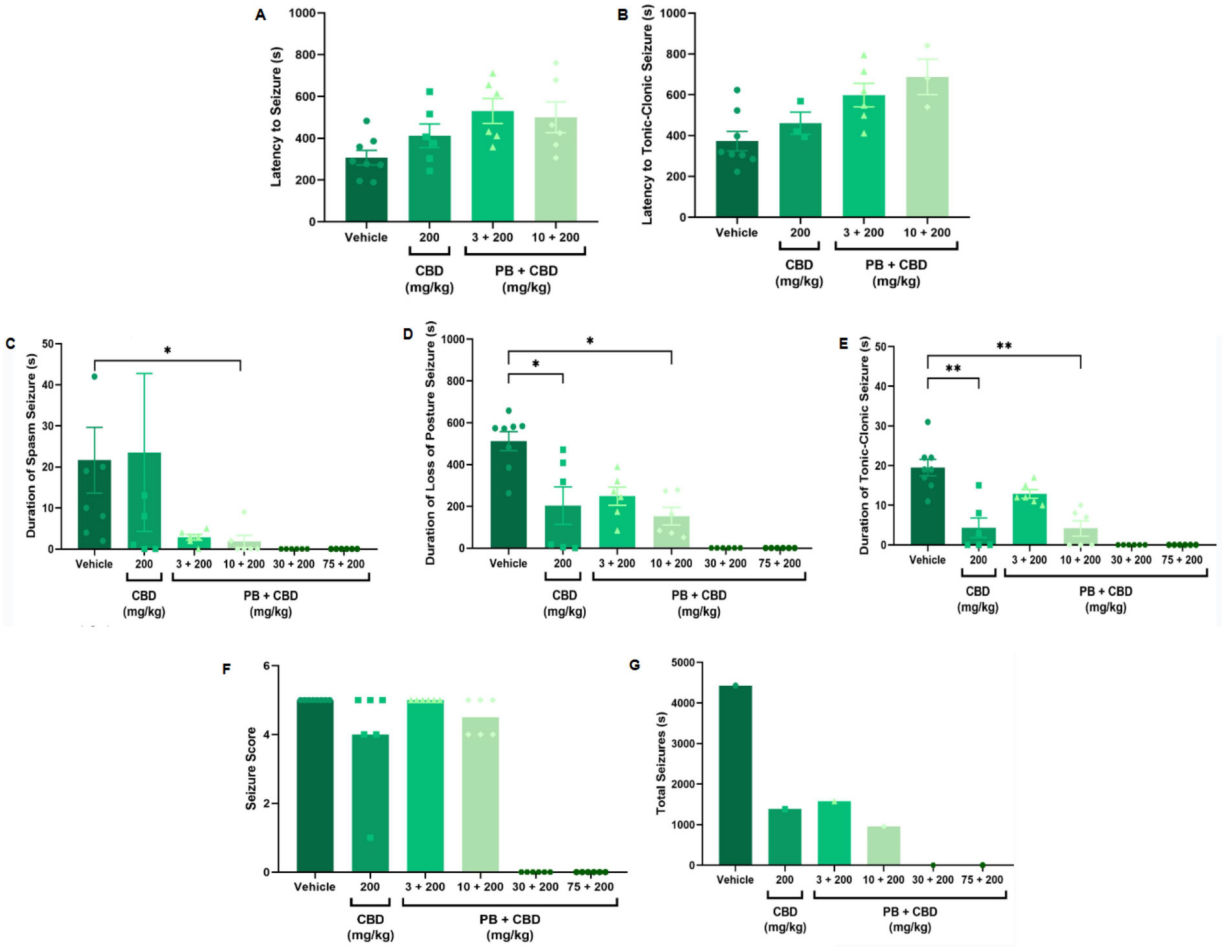
Antiseizure effects of cannabidiol (CBD, 200 mg/kg), administered alone or in combination with phenobarbital (PB; 3, 10, 30, or 75 mg/kg), in the PTZ-induced seizure model in P10 rats. **(A,B)** Latency to seizure onset and to the most severe (tonic-clonic) seizure; *n* = 8, 6, 6, 6 **(A,B)**. **(C–E)** Duration of spasms, loss of righting reflex (postural seizures), and tonic-clonic seizures; *n* = 8, 6, 6, 6, 6, 6 **(C–E)**. **(F)** Seizure severity score; *n* = 8, 6, 6, 6, 6, 6. **(G)** Total seizure duration; *n* = 8, 6, 6, 6, 6, 6. Treatment with 200 mg/kg CBD alone significantly reduced the duration of postural and tonic-clonic seizures **(D,E)**. Additionally, the combination of 10 mg/kg PB with 200 mg/kg CBD further reduced the duration of spasms, loss of righting reflex, and tonic-clonic seizures **(C–E)**. No significant reductions in seizure severity **(F)** or total seizure duration **(G)** were observed across groups, excluding the control. Data were analyzed using the Kruskal–Wallis test. *p* < 0.05 was considered statistically significant (*p = 0.0199, *p = 0.0425, *p = 0.0117; **p = 0.0038, **p = 0.0016 in respective panels).

**Figure 6:**
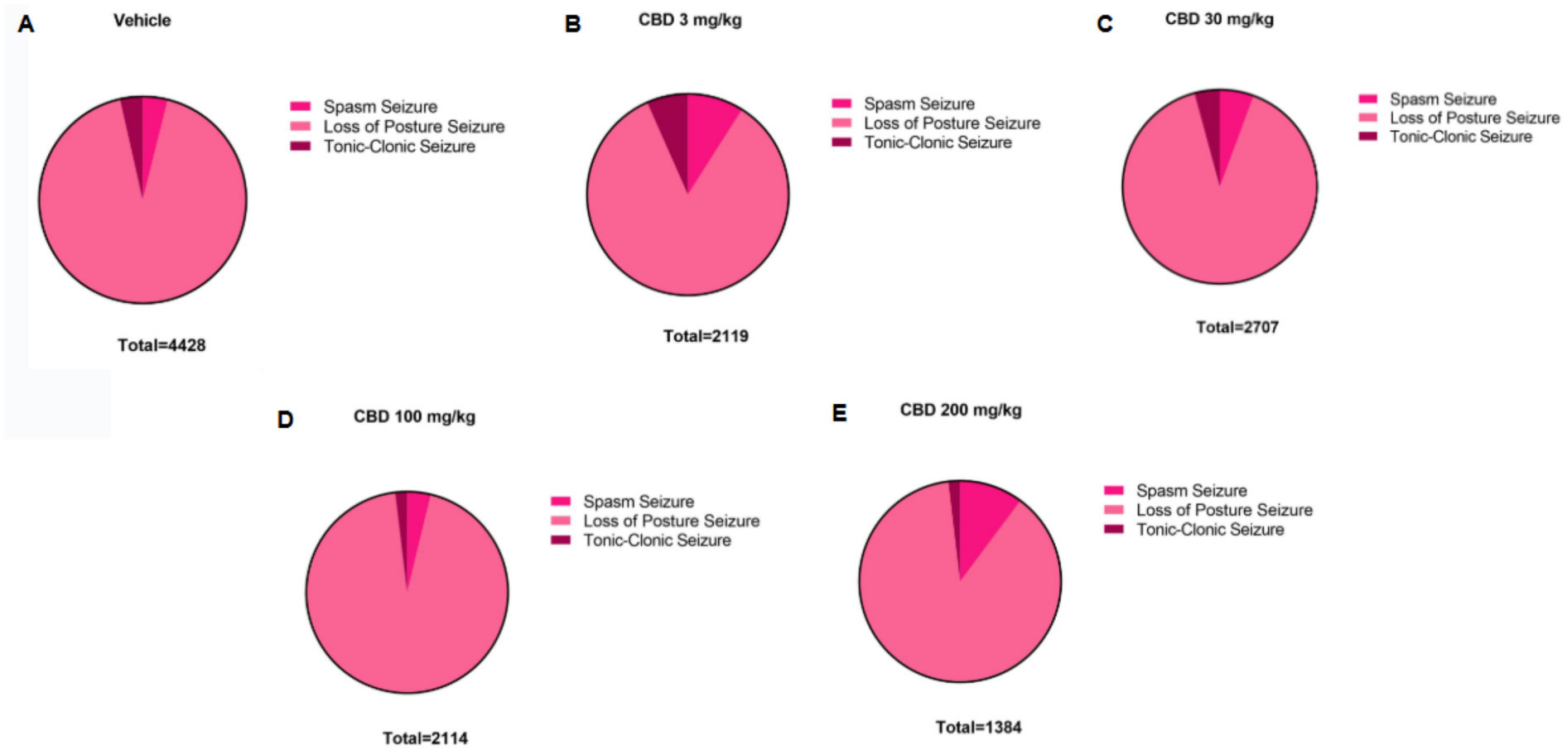
Distribution of PTZ-induced seizure activity in P10 rats following treatment with different doses of cannabidiol (CBD). Total time spent in each seizure type—spasms, loss of righting reflex (postural seizures), and tonic-clonic seizures—is shown for the the **(A)** control group (*n* = 8) and **(B–E)** animals treated with 3 (*n* = 6); 30 (*n* = 6); 100 (*n* = 6) or 200 (*n* = 6) mg/kg of CBD. Data reflect the cumulative duration of each seizure type, allowing comparison of seizure expression across different treatment groups.

To evaluate the antiseizure efficacy of PB in a neonatal model of pentylenetetrazole (PTZ)-induced seizures, postnatal day 10 (P10) rats were pretreated with varying doses of PB. In the control group (PTZ alone), seizures occurred rapidly following administration, characterized by spasms, loss of righting reflex or posture, and tonic-clonic seizures. All animals in this group reached the maximum seizure severity score of 5 (Figures 1–6, in Vehicle). These seizures were prolonged and severe, with a cumulative seizure duration of 4,428 s (s), 93% of which consisted of the “loss of righting reflex” phase. The duration of this phase ranged from 156–4,099 s, with a mean of 1,476 ± 2,272 s (Figure 6A).

To establish a dose-response profile, PB was administered at 10, 30, 50, or 75 mg/kg (Figure 2). Doses of 30 mg/kg and above fully suppressed PTZ-induced seizures, demonstrating robust antiseizure effects (Figures 2C–G). In contrast, animals treated with 10 mg/kg PB displayed seizure profiles comparable to the control group, with no significant differences in seizure latency, duration, or severity (Kruskal–Wallis, *p* > 0.05) (Figure 2). These animals exhibited a total seizure duration of 2,422 s, with approximately 88% of that time spent in the loss of righting reflex phase (range: 118–2,142 s; mean: 807.3 ± 1,156 s) (Figures 2G, 8A). Most animals in this group also reached the maximum severity score, reinforcing that 10 mg/kg is a subeffective dose under these conditions (Figure 2F).

### 3.2 Pharmacological interactions between cannabidiol (CBD) and PB in PTZ-induced seizures in neonatal rats

Although CBD has been widely explored as a potential treatment for neonatal seizures, recent studies suggest it has limited antiseizure efficacy when used as monotherapy. For example, Witherspoon et al. (41) reported that high doses of CBD (200 mg/kg) produced only modest effects in a neonatal PTZ-induced seizure model. Based on these findings, we hypothesized that CBD could potentiate the antiseizure effects of PB, particularly at subeffective doses.

To test this, we combined effective (30 and 75 mg/kg) and subeffective (3 and 10 mg/kg) doses of PB with CBD administered at 3, 30, 100, or 200 mg/kg. As expected, animals receiving PB (30 or 75 mg/kg), either alone or in combination with any dose of CBD exhibited complete protection against PTZ-induced seizures (Figures 1–4). In contrast, rats treated with low-dose PB (3 or 10 mg/kg), low-dose CBD (3 mg/kg), or their combinations displayed seizure activity comparable to the control group (Figures 1, 2).

Notably, animals treated with 3 mg/kg CBD alone exhibited a significantly shorter duration of seizure-associated loss of the righting reflex compared to those receiving 10 mg/kg PB combined with 3 mg/kg CBD (Kruskal–Wallis, H = 9.750, *p* = 0.0208; Figure 1D). Although some animals in the 3 mg/kg CBD and 3 mg/kg PB + 3 mg/kg CBD groups did not reach the maximum seizure severity score of 5, no statistically significant differences in seizure severity were detected among these groups (Kruskal–Wallis, *p* > 0.05; Figure 1F).

**Figure 7:**
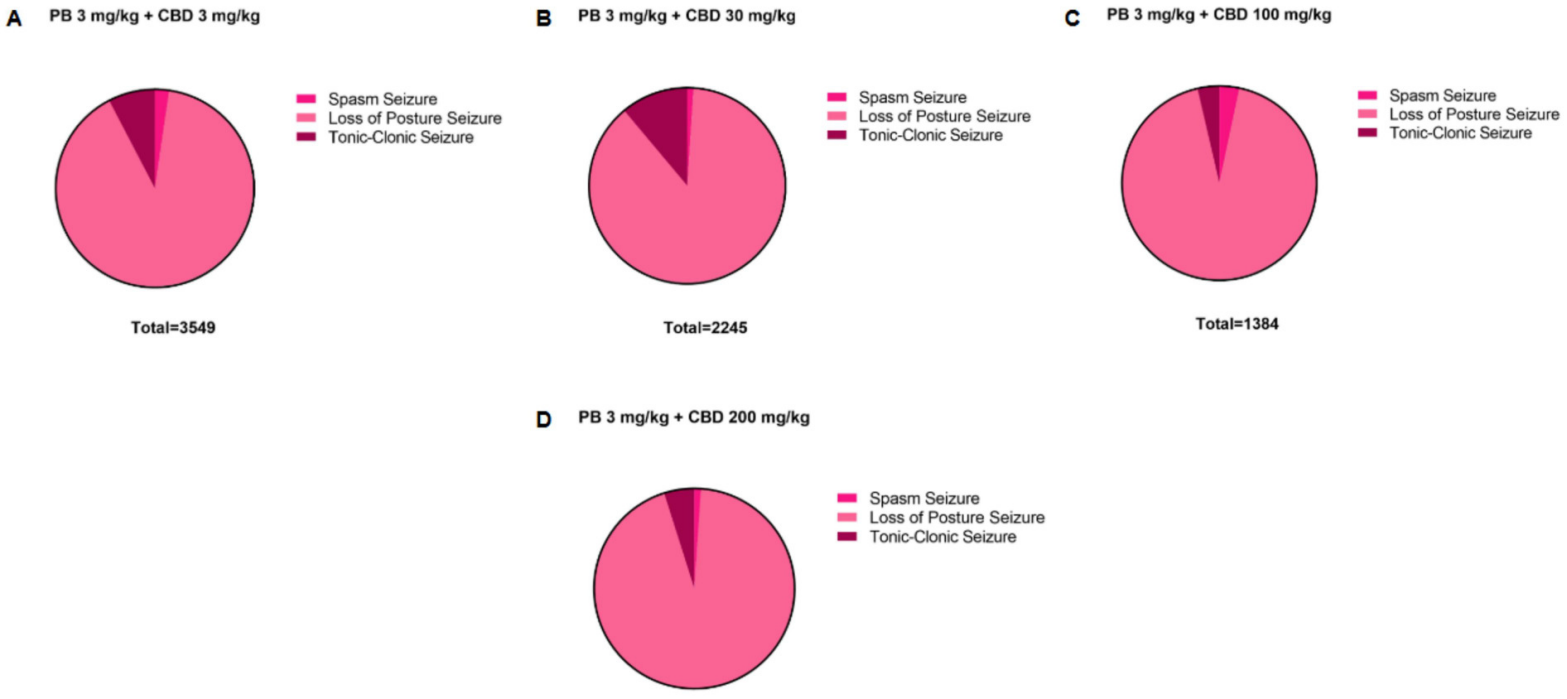
Distribution of pentylenetetrazole (PTZ)-induced seizures in P10 rats after treatment with the 3 mg/kg dose of phenobarbital (PB), together with the different doses of cannabidiol (CBD). Total time spent in each seizure by the groups treated with the 3 mg/kg PB dose, **(A)** together with the 3 mg/kg (*n* = 7), **(B)** 30 mg/kg (*n* = 6), **(C)** 100 mg/kg (*n* = 6) or **(D)** 200 (*n* = 6) mg/kg CBD doses. Seizures can be called: spasms, loss of the righting reflex or loss of posture and tonic-clonic.

**Figure 8:**
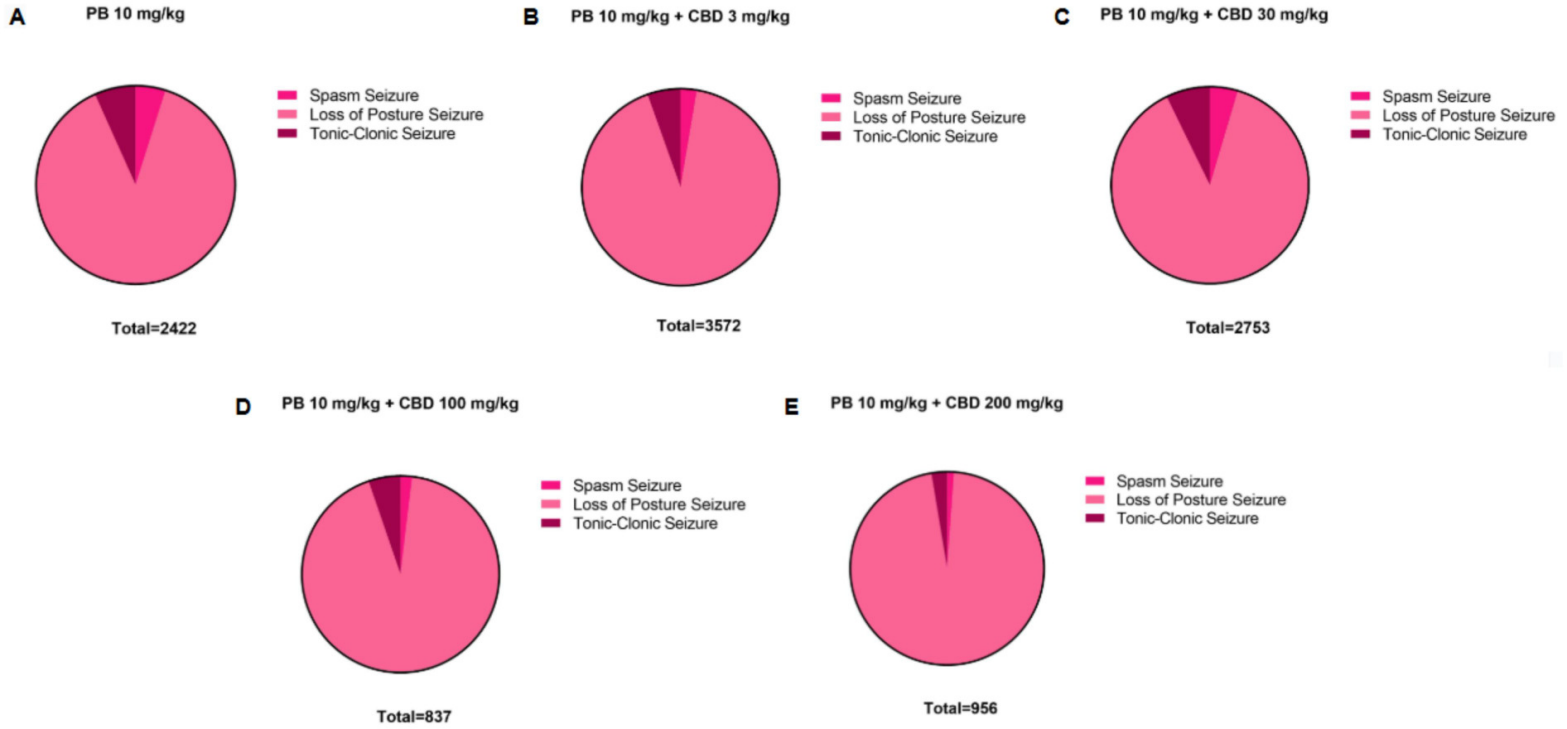
Distribution of pentylenetetrazole (PTZ)-induced seizures in P10 rats after treatment with the 10 mg/kg dose of phenobarbital (PB), alone or together with the different doses of cannabidiol (CBD). Total time spent in each seizure by the groups treated with the **(A)** 10 mg/kg PB dose, alone (*n* = 7) or **(B–E)** together with the 3 mg/kg (*n* = 6), 30 mg/kg (*n* = 10), 100 mg/kg (*n* = 6) or 200 mg/kg (*n* = 6) CBD doses. Seizures can be called: spasms, loss of the righting reflex or loss of posture and tonic-clonic.

Additionally, the group treated with 3 mg/kg CBD alone exhibited a lower total seizure duration (2,119 s) compared to the groups that received 3 mg/kg or 10 mg/kg PB combined with 3 mg/kg CBD, which displayed total seizure durations of 3,549 s and 3,572 s, respectively (Figures 1G, 6B, 7A, 8B). In the CBD-only group, approximately 84% of the seizure duration corresponded to the “loss of righting reflex” phase, with a range of 140–1,788 s and a mean duration of 706.3 ± 937.1 s (Figure 6B). In comparison, the groups treated with PB plus CBD spent approximately 90% (range of 82–3,196 s) and 92% (range of 95–3,283 s) of their total seizure durations in this same phase, with mean durations of 1,183 ± 1,746 s and 1,191 ± 1,813 s, respectively (Figures 7A, 8B).

These findings indicate that while low-dose CBD alone may modestly reduce seizure duration, it does not substantially enhance the efficacy of subeffective doses of PB. The lack of clear and apparent potentiation at these low doses of CBD suggests that higher doses may be required to achieve potentiation effects by CBD of PB.

#### 3.2.1 Dose-dependent effects of phenobarbital (PB) and cannabidiol (CBD) combinations on seizure parameters

Animals treated with 10 mg/kg of PB in combination with 30 mg/kg CBD exhibited a significantly prolonged latency to tonic-clonic seizure onset compared to the control group (Kruskal–Wallis, H = 12.14, *p* = 0.0163; Figure 2B). This combination also significantly reduced the duration of seizures involving loss of the righting reflex (Kruskal–Wallis, H = 12.72, *p* = 0.0127; Figure 2D). Despite these effects, most animals in the groups treated with CBD alone (30 mg/kg), 3 mg/kg PB, or the combination of 10 mg/kg PB + 30 mg/kg CBD still reached the maximum seizure severity score of 5 (Kruskal–Wallis, *p* > 0.05; Figure 2F).

Regarding total seizure duration, animals treated with 30 mg/kg CBD alone and those receiving 3 or 10 mg/kg PB in combination with 30 mg/kg CBD displayed similar cumulative seizure times: 2,707 s, 2,245 s, and 2,753 s, respectively (Figures 2G, 6C, 7B, 8C). In all three groups, most of the seizure time (88%–90%) was spent in the loss of righting reflex phase. The average durations of this phase were range: 114–2,440 s; mean: 902.3 ± 1,332 s for CBD alone, range: 23–1,973 s; mean: 748.3 ± 1,067 s for PB 3 mg/kg + CBD 30 mg/kg, and range: 126–2,428 s; mean: 917.7 ± 1,308 s for PB 10 mg/kg + CBD 30 mg/kg (Figures 6C, 7B, 8C).

Although low-dose CBD alone (3 mg/kg) previously showed a modest reduction in the duration of severe seizures (Figure 1D), the combination of 30 mg/kg CBD with subeffective PB (3 or 10 mg/kg) generally failed to significantly alter seizure severity or total duration. The exception was the 10 mg/kg PB + 30 mg/kg CBD group, which exhibited increased latency and reduced duration of severe seizures. These results suggest a potentiation dose-specific effect by CBD of PB, though limited to certain seizure parameters.

#### 3.2.2 Effects of high-dose cannabidiol (CBD) in combination with phenobarbital (PB)

Rats treated with 3 or 10 mg/kg PB in combination with 100 mg/kg CBD exhibited significantly prolonged latencies to seizure onset compared to the control group (Kruskal–Wallis, H = 13.10, *p* = 0.0044 and H = 34.98, *p* = 0.0009, respectively; Figures 3A, 4A). Furthermore, the combination of 10 mg/kg PB with 100 mg/kg CBD significantly reduced the duration of seizure-associated loss of the righting reflex (Kruskal–Wallis, H = 10.63, *p* = 0.0139; Figure 3D). While some animals in these groups did not reach the maximum seizure severity score, overall seizure severity did not differ significantly among groups (Kruskal–Wallis, *p* > 0.05; Figure 3F).

The total seizure duration for the group receiving 100 mg/kg CBD alone was 2,114 s, compared to 1,384 s and 837 s in the 3 mg/kg PB + 100 mg/kg CBD and 10 mg/kg PB + 100 mg/kg CBD groups, respectively (Figures 3G, 6D, 7C, 8D). In the CBD-only group, approximately 94% of seizure activity involved loss of the righting reflex, with a duration range of 40–1,994 s and a mean of 704.7 ± 1,117 s (Figure 6D). In the PB + CBD groups, this seizure type comprised 93% (46–1,288 s; mean: 461.3 ± 715.9 s) and 92% (16–777 s; mean: 279 ± 431.5 s) of total seizure time, respectively (Figures 7C, 8D). These findings suggest that co-administration of high-dose CBD with subeffective PB, particularly at 10 mg/kg, may enhance latency and reduce the duration of severe seizures.

#### 3.2.3 Effects of the highest dose of cannabidiol (CBD) (200 mg/kg)

Animals treated with 200 mg/kg CBD alone showed a significant reduction in the duration of both postural (loss of righting reflex) and tonic-clonic seizures compared to the control group (Kruskal–Wallis, H = 12.18, *p* = 0.0068 and H = 18.16, *p* = 0.0004, respectively; Figures 5D, 5E). Similarly, the combination of 10 mg/kg PB with 200 mg/kg CBD significantly decreased the duration of spasms (Kruskal–Wallis, H = 9.191, *p* = 0.0269), postural seizures (Kruskal–Wallis, H = 12.18, *p* = 0.0068), and tonic-clonic seizures (Kruskal–Wallis, H = 18.16, *p* = 0.0004) (Figures 5C–E). Although most animals in these groups did not reach the maximum seizure severity score, no statistically significant differences in severity were detected across groups (Kruskal–Wallis, *p* > 0.05; Figure 5F).

Total seizure durations were comparable across the CBD-only and PB + CBD groups: 1,384 s (CBD alone), 1,580 s (PB 3 mg/kg + CBD), and 956 s (PB 10 mg/kg + CBD) (Figures 5G, 6E, 7D, 8E). In the CBD-only group, 88% of seizure time involved loss of righting reflex (range: 26–1,217 s; mean: 461.3 ± 656.9 s; Figure 6E). In the PB + CBD groups, this proportion rose to 94% (17–1,486 s; mean: 526.7 ± 831.3 s) and 96% (11–920 s; mean: 318.7 ± 520.8 s), respectively (Figures 7D, 8E).

These results demonstrate that high-dose CBD, both alone or in combination with PB, can reduce the severity and duration of seizures in neonatal rats. Notably, the treatment with 200 mg/kg CBD produced the most consistent reduction across multiple seizure parameters, suggesting a dose-dependent potentiation effect by CBD at this highest dose.

#### 3.2.4 Effect exerted by different doses of phenobarbital (PB) and cannabidiol (CBD) on reducing or increasing the score of epileptic seizures in the PTZ model for P10 rats

Neonatal rats treated with varying doses of PB and CBD, either alone or in combination, exhibited differences in seizure severity and type following PTZ-induced seizures. Although all animals experienced seizures, the majority reached the highest severity scores (stages 4 or 5). Notably, only two animals, one receiving 3 mg/kg CBD and the other receiving 200 mg/kg CBD, exhibited milder seizure manifestations, classified as stages 1–3.

The distribution of seizure severity scores across treatment groups, presented as absolute numbers and percentages, is summarized in Table 1. Statistical analysis using the Kruskal–Wallis test revealed significant differences among groups in seizure severity (H = 41.43, *p* < 0.0001), indicating that specific treatment regimens influenced the extent of seizure expression (Table 1).

**Table 1:** Effect exerted by the isolated doses and in combination of cannabidiol (CBD) and phenobarbital (PB) on the distribution of animals in the different categories of seizures in the pentylenetetrazole (PTZ) model in P10 rats.

PB (mg/kg)	CBD (mg/kg)	No seizure		Score 1–3		Score 4		Score 5		Total (*n*)
0	0	0%	(0)	0%	(0)	0%	(0)	100%	(8)	(8)
0	3	0%	(0)	17%	(1)	0%	(0)	83%	(5)	(6)
	30	0%	(0)	0%	(0)	0%	(0)	100%	(6)	(6)
	100	0%	(0)	0%	(0)	50%	**(** **3)** **	50%	**(** **3)** **	(6)
	200	0%	(0)	17%	(1)	33%	**(** **2)** **	50%	**(** **3)** **	(6)
3	3	0%	(0)	0%	(0)	14%	(1)	86%	(6)	(7)
	30	0%	(0)	0%	(0)	0%	(0)	100%	(6)	(6)
	100	0%	(0)	0%	(0)	50%	**(** **3)** **	50%	**(** **3)** **	(6)
	200	0%	(0)	0%	(0)	0%	(0)	100%	(6)	(6)
10	0	0%	(0)	0%	(0)	0%	(0)	100%	(7)	(7)
	3	0%	(0)	0%	(0)	0%	(0)	100%	(6)	(6)
	30	0%	(0)	0%	(0)	10%	(1)	90%	(9)	(10)
	100	0%	(0)	0%	(0)	50%	**(** **3)** **	50%	**(** **3)** **	(6)
	200	0%	(0)	0%	(0)	50%	**(** **3)** **	50%	**(** **3)** **	(6)

The total number of animals per group and the number of animals that manifested the different types of seizures, following the score of 0–5 points, is shown in parentheses. Also, the percentage of animals that had each type of seizure is being expressed. The seizure scores are classified as follows: 0 = no change in behavior, 0.5 = wet dog tremors, 1 = myoclonic spasms, 2 = unilateral clonus, 3 = forelimb clonus, 4 = loss of the righting reflex or loss of posture, and 5 = tonic-clonic seizure. There were significant differences between the groups that scored 5 and the groups that obtained the scores: 0, 1–3 and 4. Kruskal–Wallis test, reference value: *p* < 0.05.

** *p* = 0.0014; *p* > 0.05.

In summary, co-administration of 100 mg/kg CBD with 3 or 10 mg/kg PB significantly increased seizure latency, indicating delayed seizure onset. Furthermore, treatment with 200 mg/kg CBD alone, as well as the combinations of 10 mg/kg PB with either 100 or 200 mg/kg CBD, led to significant reductions in both the duration and severity of PTZ-induced seizures. These findings suggest that higher doses of CBD, whether administered alone or in conjunction with subeffective PB, are effective in attenuating seizure activity in neonatal rats.

## 4 Discussion

Our findings demonstrate a dose-dependent potentiation of the antiseizure effects of PB by CBD in P10 Wistar rat pups. When administered alone, CBD did not significantly affect seizure latency, severity, or the proportion of animals exhibiting low vs. high seizure scores. However, when combined with PB, particularly at higher doses (100 mg/kg and 200 mg/kg), CBD significantly enhanced PB's efficacy, evidenced by increased latency to seizure onset, reduced seizure duration, and decreased seizure severity. Notably, the combination of 10 mg/kg PB with either 100 mg/kg or 200 mg/kg CBD reduced the incidence of Stage 5 seizures to levels like those observed with 30 or 75 mg/kg PB alone.

Although CBD is approved for certain rare pediatric epilepsies, its efficacy in neonates remains poorly understood. During early brain development, ongoing maturation of neurons and circuits can significantly alter pharmacological responses to antiseizure medications (10, 65, 66). Therefore, investigating CBD's therapeutic potential in neonates—and its possible role as an adjunct to existing therapies—is of particular importance. In this context, CBD may also offer long-term safety advantages (39). Most studies assessing CBD's antiseizure effects have been conducted in adult rodents (43, 67), leaving a gap in our understanding of its action in neonates or its interaction with other drugs like PB (41). PB is a first-line treatment for neonatal seizures (68), but its efficacy and safety at low doses in neonates remain limited.

One possible explanation for the observed potentiation by CBD of PB involves pharmacodynamic interactions at GABAergic synapses. PB acts as a positive allosteric modulator of GABA-A receptors, increasing chloride conductance and neuronal inhibition. CBD has been shown to modulate GABAergic transmission indirectly, and also to inhibit the reuptake of adenosine, a neuromodulator with anticonvulsant properties (69, 70). These overlapping pathways may contribute to the enhanced efficacy seen when both drugs are co-administered (69, 71). Although a pharmacokinetic interaction cannot be excluded, prior studies suggest that CBD may influence hepatic cytochrome P450 enzymes, potentially altering PB metabolism. However, the timing and acute nature of the experiment suggest that immediate pharmacodynamic mechanisms are more likely responsible for the observed effects.

The PTZ-induced seizure model used here is well-established and relies on non-competitive antagonism of GABA-A receptors, disrupting inhibitory neurotransmission and facilitating excitatory pathways (52, 53). PB counters this effect through positive allosteric modulation of GABA-A receptors, increasing chloride influx and neuronal hyperpolarization (72, 73). Notably, PB is more effective in preventing seizures than in terminating ongoing ones (74). Importantly, no direct pharmacokinetic interaction between PTZ and PB is expected, as PTZ neither induces nor inhibits cytochrome P450 enzymes (74). Our PB results align with previous studies (46, 54), which showed that PB was effective in reducing tonic seizures (Stage 5) in P7 rats. The higher doses required in our study may reflect differences in age (P10 vs. P7) or strain (Wistar vs. Sprague-Dawley), both known to affect seizure susceptibility (75).

CBD alone, even at high doses (3–100 mg/kg), was generally ineffective at altering seizure parameters, consistent with other neonatal studies. However, at 200 mg/kg, CBD significantly reduced both the duration of tonic-clonic seizures and loss of righting reflex. This aligns with previous findings: Uttl et al. (60) observed no effect at 10 mg/kg in P12 Wistar rats, but a 60 mg/kg dose modestly reduced seizure severity. Similarly, Witherspoon et al. (41) found that 200 mg/kg CBD increased seizure latency in P7 rats, but not severity. In older rats (P21), doses from 10–200 mg/kg increased latency, but severity remained unaffected. Other cannabinoids, such as cannabidivarin (CBDV), have shown similar profiles. Huizenga et al. (45) reported that CBDV at 100–200 mg/kg reduced seizure severity in P10 rats but did not affect latency. In P20 animals, a 200 mg/kg dose reduced severity and increased latency.

In our study, combinations of low-dose CBD (3 or 30 mg/kg) with subeffecitve PB (3 or 10 mg/kg) failed to interfere with PTZ effects, except for 30 mg/kg CBD + 10 mg/kg PB, which increased seizure latency and reduced postural seizure duration. These findings suggest a modest potentiating effect at intermediate doses. More strikingly, co-administration of high-dose CBD (100 or 200 mg/kg) with 10 mg/kg PB produced consistent antiseizure effects across several metrics, including latency, duration, and severity.

Collectively, these results underscore the importance of dose optimization. Low doses of CBD alone or in combination were largely ineffective, whereas higher doses enhanced PB's efficacy in neonatal animals. This supports the potential of CBD as an adjunctive therapy in neonatal seizure management, particularly when standard PB dosing is subeffective.

However, it is critical to highlight the need for further studies on the long-term safety and neurodevelopmental impact of CBD in the immature brain. While CBD shows promise, especially in enhancing PB's efficacy, immature neurons differ functionally from mature ones. CBD's mechanisms—potentially independent of CB1 receptor activity (43), involving modulation of ion channels (76) and non-cannabinoid receptors (77)—may operate differently in neonatal networks.

Developmental differences in receptor expression and synaptic integration (78–80) likely underlie the reduced efficacy of CBD in immature brains. Although the endocannabinoid system is present during embryogenesis (81), receptor binding capacity and functional maturation vary across brain regions and developmental stages (82, 83). This neurodevelopmental immaturity, combined with high baseline excitability, may limit CBD's capacity to modulate seizure networks during early life.

In addition, there are few studies on the control of epileptic seizures with chronic administration of CBD, especially when it comes to the neonatal period (41). Some studies involving adult models have reported that CBD, when administered chronically, is more effective when injected more than once a day, which confers a constant concentration of such a drug in the body, resulting in a longer-lasting effect (84, 85). In addition, the efficient doses of CBD, along with the period of application, suffer variations dependent on the seizure induction model. Studies with genetic models of epilepsy, such as the use of WARs (Audiogenic Wistar Rats) to perform the audiogenic kindling, a protocol that consists of the stimulation of seizures through sound, showed that animals that were treated with CBD for a period of around 12 days, receiving two injections of the drug per day at doses of 25 mg/kg, presented attenuation of brainstem seizures, such as tonic-clonic, and limbic seizures. Also, treatment with CBD was able to reduce the activity of neurons, demonstrated by the reduction of FosB immunostaining, a marker of neuronal activity, and slowed down the elevation of CB1 receptor expression in the hippocampus, normally induced by such kindling (86).

Kindling PTZ was also widely used as a model in adult rodents to test the efficacy of chronic treatment with CBD, and it was found that this drug, depending on the dose and route of administration, was able to reduce animal mortality, in addition to being able to cause an increase in the latencies of generalized seizures (87). In addition, studies that have performed chronic applications of CBD in adult rodents in the hippocampal kindling model have found that such a drug is able to reduce the progress of epileptogenesis, in addition to reducing focal and generalized seizures, and this is due to CBD acting on several receptors responsible for reducing brain electrical activity (84, 88).

Finally, studies in adult models—such as maximal electroshock seizures (MES)—show that CBD tends to exhibit antiseizure effects high doses, close do the toxic threshold (around 300 mg/kg), suggesting a narrow therapeutic range (89). Median effective doses in adults typically range from 80–90 mg/kg (90, 91). However, few studies have extended these findings to neonatal models. In PTZ- and kainate-induced seizure models, intrahippocampal CBD showed efficacy at low doses, while systemic administration was less effective (55).

## 5 Conclusion

In conclusion, this study demonstrates that CBD, particularly at high doses, can potentiate the antiseizure effects of PB in neonatal rats. Although our results support a beneficial interaction between CBD and PB in acute seizure models, the long-term consequences of high-dose CBD exposure during early brain development remain unknown. Studies have shown that certain antiseizure drugs can cause neurodevelopmental disturbances when administered during critical periods of brain maturation. Therefore, future studies should address whether co-administration of CBD and PB in neonates alters cognitive, behavioral, or neuroanatomical outcomes during adolescence or adulthood. These findings highlight the potential of CBD as an adjunct therapy for neonatal seizures, particularly when PB alone is insufficient. However, the developing brain presents unique challenges and risks. Future research should focus on elucidating the mechanisms underlying these interactions and evaluating the long-term safety of such combination therapies during early brain development.

## Data Availability

The raw data supporting the conclusions of this article will be made available by the authors, without undue reservation.
